# Patterns of Lymph Node Metastases After Multimodality Therapy in Esophageal Carcinoma Treated with Robot-Assisted Minimally Invasive Esophagectomy (RAMIE)

**DOI:** 10.1245/s10434-026-20410-z

**Published:** 2026-08-12

**Authors:** Maxime E. Sanders, Eline de Groot, Robin B. den Boer, Jan Erik Freund, Nadia Haj Mohammad, Gert J. Meijer, Stella Mook, Jelle P. Ruurda, Richard van Hillegersberg

**Affiliations:** 1 Department of Surgery, University Medical Center Utrecht, Utrecht, The Netherlands; 2 Department of Radiation Oncology, University Medical Center Utrecht, Utrecht, The Netherlands; 3 Department of Radiology, University Medical Center Utrecht, Utrecht, The Netherlands; 4 Department of Pathology, University Medical Center Utrecht, Utrecht, The Netherlands; 5 Department of Medical Oncology, University Medical Center Utrecht, Utrecht, The Netherlands

**Keywords:** Esophageal cancer, Lymph node metastases, 2-field lymphadenectomy, Tumor histology, Tumor location, Neoadjuvant therapy

## Abstract

**Background:**

Lymph node metastases (LNMs) in esophageal carcinoma demonstrate extensive and variable dissemination. Previous mapping studies are limited to region-based reporting, heterogeneous classification systems, and absence of contemporary multimodality-treated cohorts. This study aimed to characterize the station-level distribution of LNMs after standardized robot-assisted minimally invasive transthoracic esophagectomy (RAMIE) with two-field lymphadenectomy and to evaluate patterns in relation to key preoperative parameters.

**Methods:**

A prospective, single-center observational cohort of patients undergoing transthoracic RAMIE for adenocarcinoma (AC) or squamous cell carcinoma (SCC) between 2018 and 2024 was analyzed. The primary outcome was station-level distribution of LNMs in relation to histology, tumor location, clinical T stage, clinical N stage, and receipt of neoadjuvant therapy. Analyses were descriptive.

**Results:**

Among 210 patients (74% AC, 26% SCC), median lymph node yield was 44 (interquartile range [IQR], 34–52). Lymph node metastases were present in 42% of the patients, with a median of two positive nodes (IQR, 1–4). Although 91% received neoadjuvant therapy (85% CROSS, 7% FLOT), residual nodal disease remained frequent and widely distributed. Metastases occurred most frequently in the paraesophageal (11%) subcarinal (10%) mediastinal stations, and along the left gastric artery (22%) and right paracardial (15%) nodes. Thoracic duct LNM was identified in 9% of the patients. Patterns were similar across preoperative parameters. Occult nodal disease was observed in 32% of cN0 patients.

**Conclusions:**

Lymphatic dissemination in esophageal cancer remains extensive and unpredictable, even after multimodality therapy. The persistence of cross-diaphragmatic nodal involvement across subgroups underscores the ongoing necessity of a systematic two-field lymphadenectomy to achieve optimal locoregional control and accurate pathologic staging.

**Supplementary Information:**

The online version contains supplementary material available at https://doi.org/10.1245/s10434-026-20410-z.

Esophageal carcinoma ranks as the 11th most common malignancy and the 7th leading cause of cancer-related mortality worldwide.^1,2^ Despite advances in multimodal therapy, overall survival remains poor. The two predominant histologic subtypes, squamous cell carcinoma (SCC) and adenocarcinoma (AC), differ in epidemiology. Whereas SCC remains most prevalent in high-incident regions such as East Asia and parts of Africa, the incidence of AC continues to rise rapidly across Western countries, largely driven by increasing rates of obesity and gastroesophageal reflux disease.^2^

Patients typically present with progressive dysphagia, weight loss, and symptoms reflecting locally advanced disease.^3^ Lymph node metastases (LNMs) are among the strongest determinants of survival, with both the presence and number of positive nodes tightly linked to recurrence and mortality.^4–6^ The esophagus possesses an exceptionally dense and longitudinally oriented lymphatic network. Lymphatic capillaries penetrate the wall circumferentially but drain predominantly along the longitudinal axis, facilitating cranio-caudal dissemination across cervical, mediastinal, and abdominal nodal stations.^7,8^ In addition, the thoracic duct and its tributaries form a major conduit for longitudinal lymphatic flow, providing a potential pathway for cross-diaphragmatic and contralateral dissemination. Skip metastases, in which disease bypasses expected regional stations, are frequently reported.^9,10^ Consequently, predicting which nodal stations will harbor metastases is inherently difficult, and current staging modalities such as endoscopic ultrasound (EUS) and positron emission tomography/computed tomography (PET/CT) lack sufficient accuracy to identify involved nodes, particularly after neoadjuvant therapy.^11^

Curative-intent treatment for locally advanced esophageal cancer typically consists of neoadjuvant therapy followed by esophagectomy with systematic lymphadenectomy. In Western practice, multimodal treatment is guided primarily by the CROSS and ESOPEC trials.^12,13^ The CROSS trial demonstrated a significant survival benefit of neoadjuvant followed by surgery compared with surgery alone, establishing it as the standard for more than a decade in many centers.^12^ More recently, the ESOPEC trial reported superior overall survival with perioperative chemotherapy compared with neoadjuvant chemoradiotherapy for AC.^13^

Understanding the anatomic distribution of LNMs is critical for defining the appropriate extent of surgical lymphadenectomy. Inadequate dissection risks residual locoregional disease, whereas overly extensive lymphadenectomy may increase operative complexity and postoperative morbidity.^14^

Previous studies have failed to identify consistent or predictable patterns of nodal spread, largely due to heterogeneous classification systems and region-based reporting, and have predominantly focused on SCC. As a result, detailed station-level data in contemporary Western multimodality-treated populations remain limited.^15–17^ The current study aimed to provide a detailed, station-level characterization of LNMs in esophageal carcinoma after standardized robot-assisted minimally invasive transthoracic esophagectomy (RAMIE) with two-field lymphadenectomy, and to evaluate associations with key preoperative tumor characteristics including histology, tumor location, clinical T stage, and receipt of neoadjuvant therapy.

## Methods

### Study Design

This single-center observational cohort study was conducted at the University Medical Center Utrecht (UMC Utrecht). The objective was to map the locoregional distribution of LNMs in patients with esophageal carcinoma after transthoracic esophagectomy and to evaluate its association with histologic subtype, tumor location on preoperative PET/CT, clinical T stage, clinical N stage, and receipt of neoadjuvant therapy. The study adhered to the STROBE guidelines, was conducted in accordance with the Declaration of Helsinki, and was approved by the institutional review board (UMC Utrecht; number 13/061). The requirement for individual informed consent was waived in accordance with local regulations.

### Patient Selection

Patients with esophageal carcinoma who underwent transthoracic esophagectomy between November 2018 and February 2024 at UMC Utrecht were eligible. No formal sample size calculation was performed. All consecutive eligible patients were included.

### Inclusion Criteria

The study included patients if they had

- Histologically confirmed SCC or AC of the esophagus or gastroesophageal junction (GEJ)
- Esophageal carcinoma stage cT1–4, cN0–3, or cM0
- Transthoracic esophagectomy performed with intent of minimum two-field lymphadenectomy.

### Exclusion Criteria

The study ruled out

- Transhiatal esophagectomy, to ensure inclusion of only patients undergoing standardized two-field lymphadenectomy
- Aborted resections (“open-and-close” procedures), in which no esophagectomy was completed
- Interval metastasis/newly detected metastatic disease (cM1) at restaging or intraoperatively
- Unclear or indeterminate histology
- Prophylactic esophagectomy (e.g., Barrett’s esophagus without malignancy).

### Staging and Treatment

Preoperative staging included endoscopy with biopsy, EUS if indicated, and PET/CT of the neck, thorax, and abdomen. All cases were discussed by a multidisciplinary tumor board, and treatment decisions followed the Dutch national guidelines for esophageal carcinoma in accordance with the American Joint Committee on Cancer (AJCC)/Union for International Cancer Control (UICC) 8th edition staging system.^3^

Patients received either no neoadjuvant therapy, neoadjuvant chemoradiotherapy according to the CROSS regimen (carboplatin–paclitaxel with concurrent 41.4 Gy radiotherapy),^12^ or perioperative chemotherapy according to the FLOT regimen (docetaxel, oxaliplatin, leucovorin, and 5-fluorouracil).^18^ The regimen was selected by the multidisciplinary team based on tumor histology and location.

All included patients underwent RAMIE with two-field lymphadenectomy. The gastric conduit was reconstructed with an intrathoracic or cervical anastomosis, depending on tumor location and prior radiation fields.

### Lymphadenectomy

**Fig. 1:**
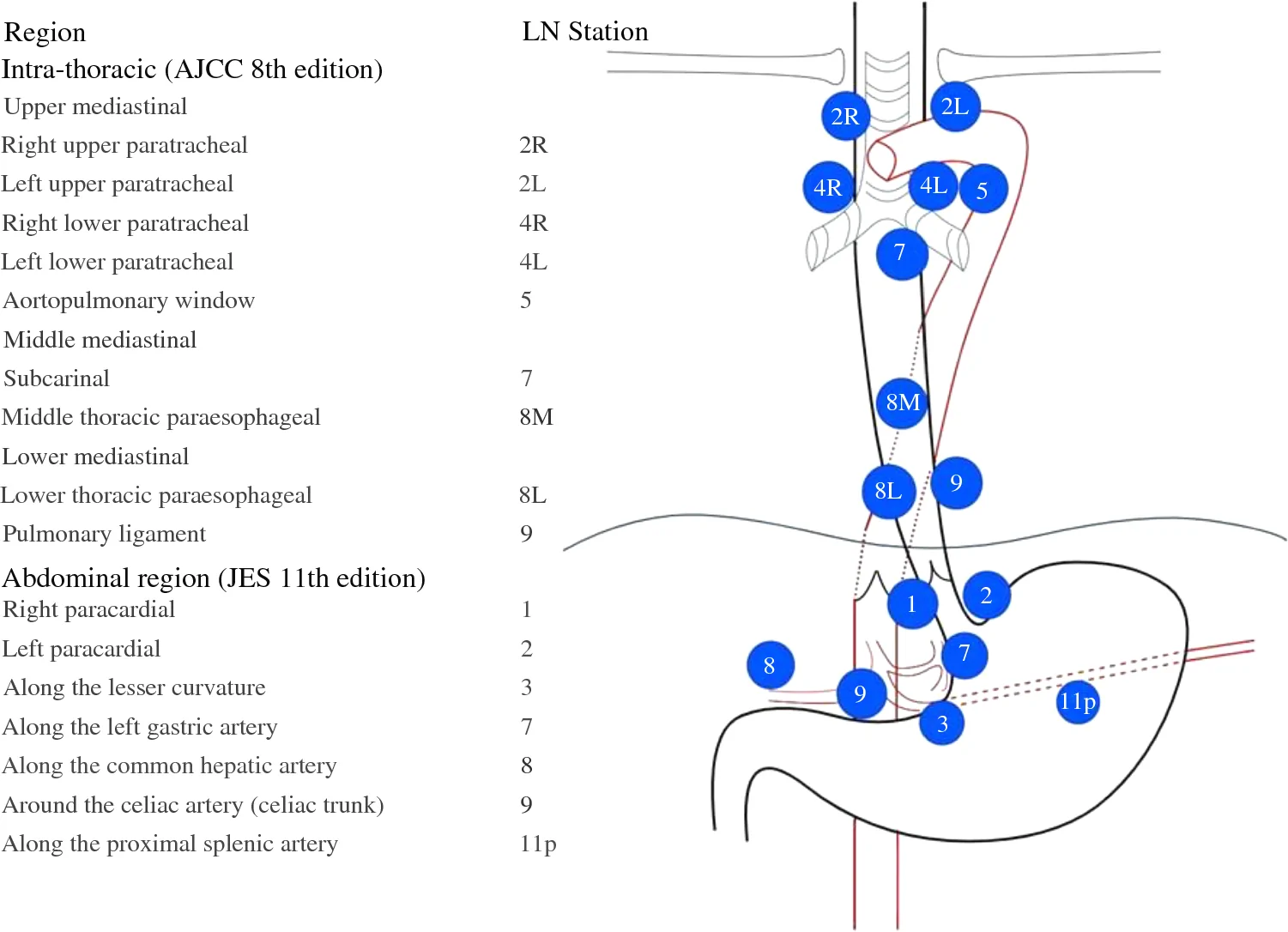
Anatomic overview of lymph node stations relevant to esophageal cancer

Lymph node stations were dissected and scored according to the AJCC 8th edition for mediastinal nodes and the Japanese Esophageal Society (JES) 11th edition for abdominal nodes, consistent with standard practice in our institution (Fig. 1). The thoracic dissection typically included paratracheal (4R/4L), aortopulmonary (5), subcarinal (7), paraesophageal (8M/8L), pulmonary ligament (9) stations and thoracic duct lymph nodes. When indicated, additional stations (e.g., anterior mediastinal (6) , tracheobronchial (10R/10L), supraclavicular, or cervical) were resected. Upper paratracheal lymph nodes (2R/2L) were dissected selectively, based on preoperative imaging or intraoperative findings. Indications for extended lymphadenectomy included suspicious nodes on preoperative imaging or intraoperative assessment. The abdominal dissection routinely included paracardial (1, 2), lesser curvature (3), left gastric artery (7), common hepatic artery (8), celiac trunk (9), and splenic hilum (11) nodes.

Nodal stations were submitted in individually labeled containers or marked by the operating surgeon for separate pathologic examination. For consistency of reporting, a lymph node station was considered “dissected” when nodal tissue from that anatomic region was retrieved and submitted as a separate, labeled specimen for pathologic evaluation. Nodal stations adjacent to the primary tumor were typically left attached to the resected specimen for margin assessment and marked accordingly.

### Histopathologic Assessment

Histopathologic examination followed the Dutch national guidelines for esophageal carcinoma.^19^ Specialized gastrointestinal pathologists microscopically examined all lymph nodes. For each nodal station, the total number of retrieved and positive nodes was recorded. For patients who received neoadjuvant therapy, nodal treatment response was recorded as complete, partial, or absent, according to institutional standards.

### Outcome

The primary aim of this study was to describe the distribution of histologically confirmed LNMs across individual lymph node stations after transthoracic esophagectomy. The distribution was analyzed in relation to key preoperative parameters: tumor histology, tumor location, clinical T stage, clinical N stage, and receipt of neoadjuvant therapy. Clinical rather than pathologic staging was used because surgical planning is based on preoperative assessment.

In addition, the overall and station-specific lymph node yield and the proportion of patients with metastases per station were reported to provide a comprehensive overview of nodal involvement patterns.

### Statistical Analysis

All statistical analyses were performed using R (version 4.3.3; R Foundation for Statistical Computing, Vienna, Austria). Continuous variables were summarized as medians with interquartile ranges (IQRs) and categorical variables as counts and percentages. The prevalence of LNMs for each lymph node station was defined as the proportion of patients with metastases in that station among those who had the station dissected. Subgroup analyses were performed according to tumor histology, tumor location, clinical T stage, clinical N stage, and receipt of neoadjuvant therapy.

Because this was a single-center study including all consecutive eligible patients treated at the study institution, there were no missing data for the primary outcomes. No multivariable analyses were performed because the primary objective was descriptive and focused on anatomic distribution patterns rather than on identifying independent predictors. Visual representations of nodal distribution were generated using R and Microsoft Excel, and schematic figures were designed using “diagrams.net (draw.io).”

Mediastinal lymph nodes are classified according to the AJCC (8th edition) and abdominal nodes according to the JES (11th edition). The schematic diagram depicts their anatomic locations alongside corresponding station numbers.

## Results

### Patient Characteristics

The study included 210 consecutive patients who underwent planned transthoracic esophagectomy at the UMC Utrecht between 5 November 2018, and 26 February, 2024. Baseline characteristics are summarized in Table 1. The cohort comprised 55 patients (26%) with SCC and 155 patients (74%) with AC. The median age was 67 years (IQR, 61–72 years), and 74% were male. Tumors were most commonly located in the distal esophagus (71%), followed by the GEJ (12%). Most patients had clinical T3 disease (65%), and 55% were clinically node-positive (cN+) at diagnosis.

**Table 1:** Baseline characteristics

Characteristic	Total *n* (%)	SCC *n* (%)	AC *n* (%)
	*n* = 210 (100)	*n* = 55 (26)	*n* = 155 (74)
Median age: years (IQR)	67 (61–72)	69 (62–72)	66 (60–72)
*Gender*			
Male	156 (74)	24 (44)	132 (85)
Female	54 (26)	31 (56)	23 (15)
*Tumor location*			
Proximal esophagus	5 (2)	5 (9)	0 (0)
Middle esophagus	30 (14)	24 (44)	6 (4)
Distal esophagus	149 (71)	24 (44)	125 (81)
Gastro-esophageal junction	26 (12)	2 (4)	24 (16)
*Clinical T stage*			
cT1	9 (4)	2 (4)	7 (5)
cT2	32 (15)	9 (16)	23 (15)
cT3	137 (65)	24 (44)	113 (73)
cT4	32 (15)	20 (36)	12 (8)
*Clinical N stage*			
cN0	95 (45)	24 (44)	71 (46)
cN+	115 (55)	31 (56)	87 (54)
cN1	78 (37)	20 (36)	61 (37)
cN2	25 (12)	6 (11)	15 (12)
cN3	12 (6)	5 (9)	12 (5)
*Neoadjuvant therapy*			
None	18 (9)	6 (11)	12 (8)
Yes	192 (91)	49 (89)	146 2)
CTRX	178 (85)	49 (89)	129 (83)
CTX	14 (7)	0 (0)	14 (9)

SCC, squamous cell carcinoma; AC, adenocarcinoma; IQR, inter-quartile range; CTRX, chemoradiotherapy; CTX, chemotherapy

Preoperative therapy was administered to 192 patients (91%), predominantly chemoradiotherapy according to the CROSS regimen (85%), whereas 7% received chemotherapy (FLOT) and 9% underwent primary surgery without neoadjuvant therapy. As expected, SCC was more frequently located in the middle esophagus (44%), whereas AC predominantly occurred in the distal esophagus or GEJ (97%).

### Lymphadenectomy and Lymph Node Yield

A total of 9292 lymph nodes were resected, with a median yield of 44 (IQR, 34–52) nodes per patient. The prevalence of nodal metastasis was 42% (88/210). Among the patients with nodal involvement, the median number of positive nodes was 2 (IQR, 1–4) (Table 2).

**Table 2:** Lymph node (LN) yield

	Total(*n* = 210)	SCC(*n* = 55)	AC(*n* = 155)
Patients with LNM: *n* (%)	88 (42)	19 (35)	69 (45)
*Lymph node yield*			
Median (IQR)	44 (34–52)	44 (34–52)	44 (35–51)
Range	16–87	16–77	17–87
*Positive LN per patient*^*a*^			
Median	2	1	2
Range	1–23	1–4	1–23

SCC, squamous cell carcinoma; AC, adenocarcinoma; LNM, lymph node metastasis; IQR, interquartile range

^a^Among patients with at least one positive node

When stratified by histology, metastases were identified in 35% of SCC and 45% of AC cases. The overall nodal yield and number of positive nodes did not differ notably between histologic subtypes. Detailed nodal yield per station is summarized in Table S1. Lymph nodes were retrieved from the right paratracheal (2R) station in 43% and from the left paratracheal (2L) station in 20% of cases, reflecting the selective nature of upper paratracheal dissection. Among the 64 patients with a complete pathologic response of the primary tumor (ypT0), 13 (20%) nonetheless harbored residual nodal metastases (ypN+).

### Distribution of Lymph Node Metastases

**Fig. 2:**
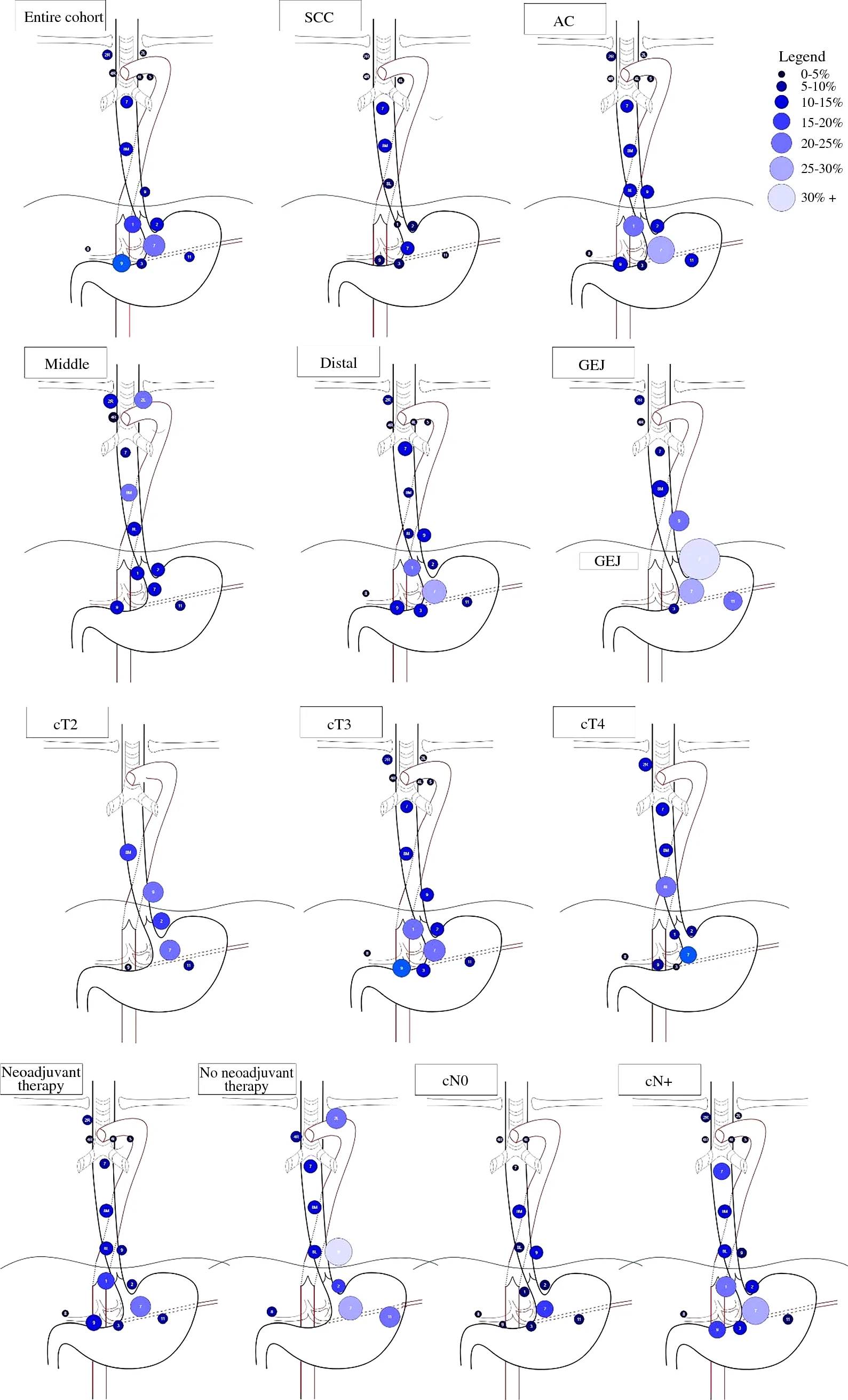
Distribution and frequency of lymph node metastases by tumor characteristics

The spatial distribution of nodal metastases is shown in Fig. 2, Table 3, and Tables S2 and S3. Prevalence per station was defined as the proportion of patients with metastases among those who had that station dissected. Subgroup analyses were performed according to tumor histology, location, clinical T and N stages, and neoadjuvant therapy. Proximal tumors (*n *= 5) and cT1 disease (*n *= 9) were excluded from schematic visualization due to limited sample size.

**Table 3:**
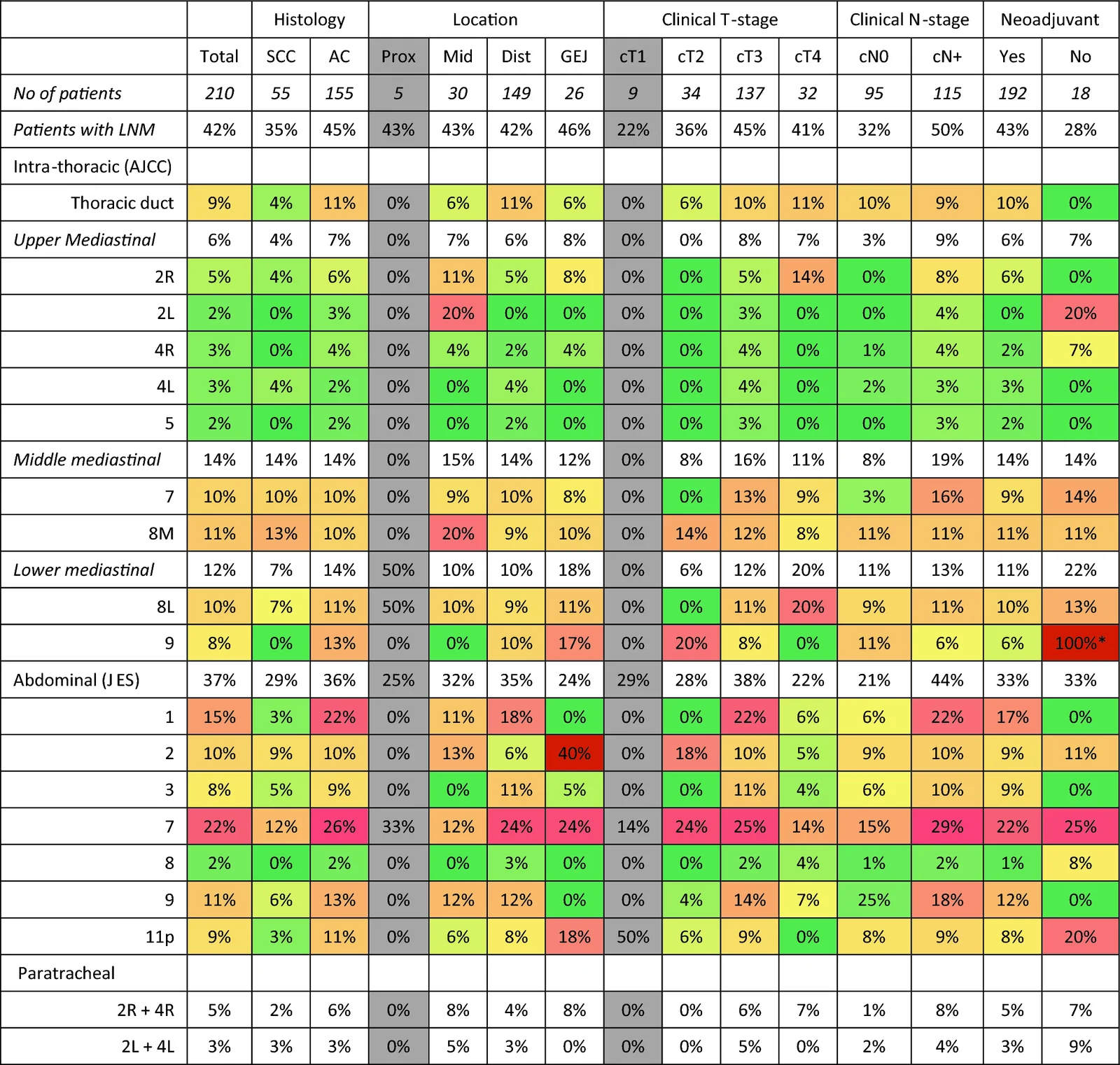
Prevalence of lymph node metastases (LNMs) per station and subgroup^a^

SCC, squamous cell carcinoma; AC, adenocarcinoma; Mid, middle; Dist, distal; GEJ, gastroesophageal junction; AJCC, American Joint Committee on Cancer; JES, Japanese Esophageal Society

^a^Cells are color-coded according to the proportion of patients with metastases in each lymph node station, ranging from green (low prevalence) to red (high prevalence). Gray-shaded cells indicate that fewer than 15 patients were included in the respective subgroup. Aggregated regional categories (e.g., all abdominal, upper mediastinal) represent summary groupings and are consequently left uncolored in the table.

^b^Percentages may appear high in small subgroups

Across the cohort, mediastinal involvement was most frequent in the middle (8M, 11%), lower paraesophageal (8L, 10%), and subcarinal (7, 10%) stations, followed by the pulmonary ligament (9, 8%). Paratracheal nodal involvement was observed in stations 2R (5%), 2L (2%), 4R (3%), and 4L (3%). In the abdomen, metastases most commonly involved the left gastric artery (7, 22%), right paracardial (1, 15%), celiac trunk (9, 11%), and left paracardial (2, 10%) stations.

### Histology

Both tumor types demonstrated bidirectional lymphatic spread across thoracic and abdominal regions. In SCC, nodal involvement was most frequent in the middle paraesophageal (8M, 13%), left gastric artery (7, 12%), and subcarinal (7, 10%) stations, with paratracheal metastases in stations 2R (4%) and 4L (4%). Adenocarcinoma showed a marked abdominal predominance, with the highest prevalence along the left gastric artery (7, 26%), right paracardial (1, 22%), celiac trunk (9, 13%), and splenic artery (11p, 11%). Paratracheal involvement was observed (2R, 6%; 2L, 3%; 4R, 4%; and 4L, 2%).

### Tumor Location

Middle and distal esophageal tumors exhibited widespread nodal dissemination involving both mediastinal and abdominal fields. In middle tumors, upper paratracheal stations (2R, 11% and 2L, 20%) were more often involved than lower paratracheal stations (4R, 4% and 4L, 0%). Upper paratracheal involvement was notably higher in middle tumors than in distal or GEJ tumors, most strikingly at station 2L (middle, 20%, distal, 0%, GEJ, 0%) (Table 3). Distal tumors demonstrated extensive abdominal involvement, particularly at the left gastric artery (7, 24%), right paracardial (1, 18%), and celiac trunk (9, 12%) stations.

Gastroesophageal junction tumors showed nodal metastases above and below the diaphragm, with frequent involvement of middle and lower mediastinal as well as abdominal nodes. Right paratracheal metastases also were commonly observed (upper right (2R, 8%) and lower right (4R, 4%)) stations, whereas no left paratracheal LNMs were identified (2L, 0% and 4L, 0%).

When stratified by pathologic T stage, nodal involvement increased in frequency with advancing depth of invasion. However, metastatic spread remained distributed across both mediastinal and abdominal stations, including spread in patients with complete primary tumor regression (ypT0) (Table S3).

### Clinical T Stage

The overall prevalence of nodal metastases was 36% in cT2, 45% in cT3, and 41% in cT4 disease, indicating a stable frequency across clinical T stages. Nodal involvement in cT2–3 tumors was mainly abdominal, particularly at the left gastric artery and paracardial stations, whereas cT4 tumors more often showed mediastinal involvement, including subcarinal and paraesophageal stations. In patients with cT1 tumors, predominantly distal AC, LNM was confined to abdominal stations. Across all clinical T stages, nodal spread involved both mediastinal and abdominal stations, indicating that tumor depth alone does not determine the anatomic extent of lymphatic dissemination.

### Clinical N Stage

Pathologic LNMs were detected in 32% of cN0 and 50% of cN+ patients. Although cN+ disease was associated with a higher nodal burden, the LNM distribution overlapped substantially, with both mediastinal and abdominal involvement. Among clinically node-negative patients, occult metastases occurred most frequently along the left gastric artery (7, 15%), middle paraesophageal (8M, 11%), and pulmonary ligament (9, 11%) stations.

### Neoadjuvant Therapy

Of the 210 patients, 192 (91%) received neoadjuvant therapy and 18 (9%) underwent upfront surgery. The upfront surgery group consisted predominantly of early-stage tumors: 9 of 18 (50%) were cT1, 7 of 18 (39%) were cT2, and 2 of 18 (11%) were cT3. Most were clinically node-negative (16/18, 89%) and included 6 SCCs (33%) and 12 ACs (67%). Pathologic nodal metastases were identified in 18% of these patients.

Among patients treated with neoadjuvant therapy, nodal metastases were present in 43%. Patterns of spread were similar to those seen in the overall cohort, with involvement across both mediastinal and abdominal stations. A small subset of patients received perioperative FLOT chemotherapy (*n *= 14), comprising distal (*n *= 6) and GEJ (*n *= 8) tumors. In this group, 57% harbored residual nodal metastases. Metastatic involvement was observed in both thoracic and abdominal fields, with the majority located in abdominal stations. Thoracic duct metastases were identified in 18%.

## Discussion

This study provides a contemporary, station-level characterization of LNM in esophageal carcinoma after transthoracic esophagectomy with standardized two-field lymphadenectomy. In a consecutive cohort of 210 patients treated predominantly with neoadjuvant therapy, lymphatic spread was frequent and widely distributed, involving nodal stations across both mediastinal and abdominal regions. Both AC and SCC demonstrated extensive cross-diaphragmatic dissemination, although with expected regional tendencies, with SCC more often involving mediastinal stations related to the higher esophageal tumor location and AC showing a predominance of abdominal nodal disease.

Despite these differences in emphasis, the overall pattern of spread was broadly similar across preoperative parameters. Occult nodal disease was common, and residual LNMs were identified even in patients with complete pathologic tumor regression. Collectively, these findings indicate that lymphatic dissemination in the modern multimodality era remains widespread and cannot be reliably predicted based on preoperative characteristics or staging alone.

The distribution observed in this cohort aligns with previous mapping studies, which have consistently shown a widespread and bidirectional lymphatic dissemination in esophageal carcinoma involving both mediastinal and abdominal stations.^17,20–25^ However, most of these analyses reported nodal involvement only at the regional level rather than the station level, based largely on open or transhiatal esophagectomy, and predated contemporary neoadjuvant protocols. A comprehensive systematic review highlighted major limitations of the existing literature, including heterogeneous node classification systems, limited station-level reporting, and a predominance of SCC cohorts. This restricts the generalizability of these findings to Western AC-dominant populations.^26^ By delivering high-resolution mapping under uniform RAMIE conditions in a predominantly AC cohort, this study adds contemporary Western data to a field previously dominated by SCC-based or region-level analyses.

Tumor location was associated with the involvement of particular nodal stations but did not limit lymphatic spread to any single anatomic region. Middle-thoracic tumors showed higher rates of upper mediastinal involvement, most notably at paratracheal stations 2R and 2L, whereas distal esophageal tumors demonstrated predominantly abdominal metastases. Gastroesophageal junction tumors exhibited broad distribution, with nodal metastases present across both lower mediastinal and abdominal basins, particularly in the perigastric region. Notably, GEJ tumors in this series demonstrated right-sided paratracheal involvement but no left-sided LNMs, a pattern also previously reported.^17,27^ These observations support prior reports that tumor height influences the probability but not the extent, of lymphatic dissemination.^17,26,27^ Accordingly, comprehensive two-field lymphadenectomy remains appropriate across tumor sites, although highly consistent anatomic patterns, such as the absence of left paratracheal involvement in GEJ tumors, may inform future selective approaches.

Both SCC and AC exhibited extensive, bidirectional lymphatic dissemination, consistent with the longitudinal lymphatic drainage of the esophagus. Although SCC showed relatively greater mediastinal involvement and AC a stronger abdominal predominance, substantial overlap existed across nodal regions. These findings mirror prior observations that histology influences the probability of involvement in specific basins but does not confine metastases to them.^15,21,24^

Clinical T stage showed no clear association with the frequency or distribution of LNMs, indicating that tumor depth alone is an unreliable surrogate for the extent of lymphatic dissemination. Lymphatic spread appears to occur early and in a multidirectional pattern across mediastinal and abdominal basins.^28,29^ Occult nodal disease also remained frequent, with 32% of clinically node-negative (cN0) patients harboring pathologic metastases, consistent with prior work showing limited sensitivity of EUS and PET/CT for regional staging and for detecting complete response after chemoradiotherapy.^11,30–33^ The substantial overlap in nodal distribution between the cN0 and cN+ groups reinforces that clinical nodal status should not guide the extent of lymphadenectomy.

In this cohort, thoracic duct and surrounding lymph nodes were routinely dissected, and thoracic duct node involvement was identified in 9% of patients, with similar prevalences across preoperative characteristics. Although the value of routine thoracic duct dissection remains debated, involvement of these nodes is well described in anatomic and pathologic studies, reflecting the thoracic duct’s role as a major longitudinal lymphatic conduit.^34^ Prior series have reported metastatic rates ranging from 2 to 15%, often at or cranial to the primary tumor level.^15,35^ Although the therapeutic value of routine thoracic duct dissection remains debated, these findings support consideration of the thoracic duct-associated pathway as a relevant route of metastatic spread.

Despite widespread use of neoadjuvant therapy in this cohort, residual nodal disease remained frequent and widely distributed, indicating that current systemic regimens do not consistently sterilize lymphatic basins. Data from the ESOPEC trial showed that the survival advantage of perioperative FLOT over CROSS was driven by improved systemic control. Distant recurrences were markedly reduced (31.5% vs 47.2%), whereas locoregional recurrence rates were nearly identical between regimens.^36^ These findings indicate that current systemic regimens primarily influence distant disease, not regional nodal sterilization, underscoring the continued importance of thorough surgical lymphadenectomy.

Several studies have demonstrated an association between higher lymph node yield and improved survival in esophageal carcinoma, commonly attributed to more accurate staging and improved locoregional disease control.^37,38^ In this context, the high median nodal yield in the current study supports the oncologic thoroughness of the standardized two-field lymphadenectomy and the reliability of the observed station-level distribution patterns. These findings complement ongoing international initiatives such as the TIGER study, which aims to define risk-adapted lymphadenectomy strategies across tumor types and geographic regions.^39^ Until results from large multicenter efforts become available, detailed single-center datasets such as in the current study provide valuable reference material for surgical planning. However, the interpretation of LN yield as a surrogate for oncologic quality warrants caution. Emerging evidence suggests that tumor immunobiology may confound this relationship: more immunogenic tumors may drive higher nodal yields while conferring better prognosis independent of surgical thoroughness, a form of reverse causality that could overestimate the survival benefit of extended lymphadenectomy.^40^ These considerations lie beyond the scope of the current dataset but are relevant when yield-survival associations in esophageal carcinoma are interpreted.

Limitations of this study include its single-center design, modest subgroup sizes, and a cohort predominantly treated with neoadjuvant CROSS therapy, which may limit external generalizability. Although AC and SCC represent biologically distinct entities, they were analyzed within a single cohort to allow comprehensive anatomic mapping across the full spectrum of esophageal carcinoma treated in Western practice. Stratified analyses were performed throughout, with all these available in supplementary files. The analyses were descriptive by design, and no multivariable modeling was performed. Furthermore, the lymph node yields and dissection extent reported reflect a standardized technique performed at a high-volume expert center. Complication risks associated with thorough two-field lymphadenectomy may be higher in lower-volume settings, and the observed LN burden may not be directly generalizable.

Histopathologic assessment after neoadjuvant therapy introduces inherent challenges. Treatment-induced fibrosis or complete nodal regression may obscure residual micrometastatic disease, increasing the risk of false-negative nodal evaluation. Conversely, sampling error remains possible even in untreated nodes. Additionally, a small subset of peritumoral stations, particularly mediastinal stations 8L/9 and abdominal stations 1/2, were occasionally left attached to the specimen to preserve margin orientation and were not submitted as separately labeled stations and may reduce granularity for these specific locations. Furthermore, stations 2R and 2L were dissected only selectively, and thus the prevalence of LNM might be overstated. Finally, survival outcomes were not yet mature at the time of analysis, precluding correlations between nodal distribution and long-term prognosis.

Future research should focus on integrating station-level mapping with imaging, radiomic, and molecular predictors to support individualized lymphadenectomy strategies. Multicenter pooling and collaboration with international datasets are essential to validate these patterns and define prognostic thresholds. Ultimately, combining anatomic mapping with biologic and imaging biomarkers may enable selective approaches without compromising oncologic safety, whereas longitudinal analyses will clarify how evolving systemic neoadjuvant regimens influence spatial patterns of residual and metastatic disease.

## Conclusion

This study provides contemporary, station-level evidence that lymphatic dissemination in esophageal carcinoma remains extensive after multimodality treatment. The persistence of occult and residual nodal disease underscores the limited reliability of preoperative staging to predict nodal involvement. These findings support the continued role of systematic two-field lymphadenectomy to achieve accurate pathologic staging and comprehensive locoregional control.

## Supplementary Information

Below is the link to the electronic supplementary material.

Supplementary file1 (DOCX 45 KB)
