# Supplementary material for: Patterns of Lymph Node Metastases After Multimodality Therapy in Esophageal Carcinoma Treated with Robot-Assisted Minimally Invasive Esophagectomy (RAMIE)
Source: Ann Surg Oncol. 2026 Aug 12;33(12):10335–45. doi: 10.1245/s10434-026-20410-z (PMC13675271; doi:10.1245/s10434-026-20410-z)
Supplement: Supplementary file 1 [file 10434_2026_20410_MOESM1_ESM.docx]

**Table S1.** Lymph node yield and dissection frequency per nodal station.

| **Lymph node station** | **Lymph nodes resected** | | **Tumor-positive lymph nodes** | | **Station dissected with LNs** | | **Patients with LNM** | |
| --- | --- | --- | --- | --- | --- | --- | --- | --- |
|  | *Median* | *Range* | *Median* | *Range* | *n* | *%* | *n* | *%* |
| Total | 44 | 16-87 | 2 | 0-20 | 210 | 100 | 88 | 42 |
| **Intra-thoracic (AJCC)** |  |  |  |  |  | 0 |  |  |
| Thoracic duct | 1 | 0-8 | 1 | 0-3 | 111 | 53 | 10 | 9 |
| *Upper Mediastinal* |  |  |  |  | 191 | 91 | 12 | 6 |
| 2R | 3 | 0-14 | 1 | 0-9 | 91 | 43 | 5 | 5 |
| 2L | 1 | 0-6 | 1 | 0-1 | 42 | 20 | 1 | 2 |
| 4R | 7 | 0-33 | 2 | 0-6 | 178 | 85 | 5 | 3 |
| 4L | 1.5 | 0-10 | 1 | 0-3 | 114 | 54 | 3 | 3 |
| 5 | 1 | 0-6 | 1 | 0-1 | 62 | 30 | 1 | 2 |
| *Middle mediastinal* |  |  |  |  | 181 | 86 | 25 | 14 |
| 7 | 5 | 0-25 | 1 | 0-7 | 165 | 79 | 16 | 10 |
| 8M | 2 | 0-16 | 1 | 0-3 | 92 | 44 | 10 | 11 |
| *Lower mediastinal* |  |  |  |  | 101 | 48 | 12 | 12 |
| 8L | 2 | 0-17 | 1 | 0-5 | 91 | 43 | 9 | 10 |
| 9 | 1 | 0-31 | 2 | 0-6 | 36 | 17 | 3 | 8 |
| **Abdominal (JES)** |  |  |  |  | 193 | 92 | 64 | 33 |
| 1 | 2 | 0-13 | 2 | 0-6 | 85 | 40 | 13 | 15 |
| 2 | 2 | 0-9 | 1 | 0-3 | 95 | 45 | 9 | 9 |
| 3 | 2 | 0-18 | 1 | 0-7 | 127 | 60 | 10 | 8 |
| 7 | 4 | 0-21 | 1 | 0-15 | 161 | 77 | 36 | 22 |
| 8 | 3 | 0-14 | 1 | 0-3 | 169 | 80 | 3 | 2 |
| 9 | 2 | 0-12 | 1 | 0-4 | 128 | 61 | 14 | 11 |
| 11 | 1 | 0-9 | 1 | 0-2 | 103 | 49 | 9 | 9 |
| **Paratracheal** |  |  |  |  |  |  |  |  |
| 2R + 4R | 8 | 0-44 | 1 | 0-9 | 189 | 90 | 9 | 5 |
| 2L + 4L | 1 | 0-10 | 1 | 0-3 | 131 | 62 | 4 | 3 |

**Legend:**
This table summarizes the total number of lymph nodes (LNs) resected, positive LNs identified, and the frequency of dissection for each nodal station according to the **AJCC 8th edition** (intrathoracic) and **JES 11th edition** (abdominal) classification systems. Values are presented as medians with ranges. A station was classified as ‘dissected’ when lymphatic tissue from that region was retrieved and submitted separately for pathological assessment. Lower percentages may reflect the absence of distinct nodal tissue or en bloc submission near the primary tumor rather than omission from the surgical field.
The “Patients with LNM” columns indicate the number and percentage of patients with metastases in that station among those dissected.
Combined paratracheal categories (2R + 4R, 2L + 4L) summarize cumulative values for right- and left-sided stations, respectively.

**Table S2.** Prevalence of lymph node metastases (LNM) per station and clinical subgroup in patients with esophageal adenocarcinoma.

|  | **Histology** | **Location** | | | | **Cliniacl T-stage** | | | | **Clincial N-stage** | | **Neoadjuvant** | |
| --- | --- | --- | --- | --- | --- | --- | --- | --- | --- | --- | --- | --- | --- |
|  | AC | Prox | Mid | Dist | GEJ | cT1 | cT2 | cT3 | cT4 | cN0 | cN+ | Yes | No |
| No. Of patients | n=115 | n=0 | n=6 | n=125 | n=24 | n=7 | n=23 | n=113 | n=12 | n=71 | n=84 | n=143 | n=12 |
| Total | 45 |  | 50 | 43 | 50 | 29 | 39 | 45 | 58 | 34 | 54 | 45 | 42 |
| **Intra-thoracic (AJCC)** |  |  |  |  |  |  |  |  |  |  |  |  |  |
| Thoracic duct | 11 |  | 0 | 12 | 6 | 0 | 9 | 10 | 22 | 9 | 12 | 12 | 0 |
| *Upper Mediastinal* | 7 |  | 17 | 6 | 9 | 0 | 0 | 8 | 9 | 5 | 9 | 7 | 10 |
| 2R | 6 |  | 0 | 6 | 10 | 0 | 0 | 6 | 20 | 0 | 10 | 7 | 0 |
| 2L | 3 |  | 100 | 0 | 0 | 0 | 0 | 4 | 0 | 0 | 7 | 0 | 33 |
| 4R | 4 |  | 25 | 3 | 5 | 0 | 0 | 5 | 0 | 2 | 6 | 3 | 11 |
| 4L | 2 |  | 0 | 3 | 0 | 0 | 0 | 3 | 0 | 2 | 2 | 2 | 0 |
| 5 | 2 |  | 0 | 3 | 0 | 0 | 0 | 4 | 0 | 0 | 5 | 2 | 0 |
| *Middle mediastinal* | 14 |  | 25 | 14 | 13 | 0 | 6 | 17 | 9 | 8 | 20 | 14 | 22 |
| 7 | 10 |  | 25 | 9 | 9 | 0 | 0 | 13 | 0 | 4 | 15 | 9 | 22 |
| 8M | 10 |  | 50 | 9 | 11 | 0 | 11 | 9 | 33 | 9 | 11 | 9 | 20 |
| *Lower mediastinal* | 14 |  | 20 | 13 | 20 | 0 | 8 | 15 | 50 | 13 | 16 | 12 | 33 |
| 8L | 11 |  | 20 | 10 | 13 | 0 | 0 | 13 | 50* | 9 | 14 | 10 | 20 |
| 9 | 13 |  | 0 | 11 | 20 | 0 | 20 | 11 | 0 | 13 | 11 | 9 | 100 |
| **Abdominal (JGCA)** | 36 |  | 50 | 38 | 26 | 40 | 33 | 37 | 33 | 23 | 48 | 35 | 50 |
| 1 | 22 |  | 33 | 23 | 0 | 0 | 0 | 26 | 33 | 10 | 30 | 24 | 0 |
| 2 | 10 |  | 25 | 5 | 50* | 0 | 40 | 6 | 17 | 11 | 8 | 9 | 20 |
| 3 | 9 |  | 0 | 10 | 6 | 0 | 0 | 10 | 14 | 8 | 10 | 10 | 0 |
| 7 | 26 |  | 20 | 27 | 26 | 20 | 28 | 27 | 22 | 19 | 34 | 25 | 38 |
| 8 | 2 |  | 0 | 3 | 0 | 0 | 0 | 2 | 8 | 2 | 3 | 2 | 11 |
| 9 | 13 |  | 0 | 15 | 0 | 0 | 7 | 16 | 0 | 0 | 22 | 14 | 0 |
| 11p | 11 |  | 0 | 9 | 20 | 100 | 9 | 9 | 0 | 11 | 10 | 9 | 40 |
| **Paratracheal** |  |  |  |  |  |  |  |  |  |  |  |  |  |
| 2R + 4R | 6 |  | 20 | 4 | 9 | 0 | 0 | 7 | 10 | 2 | 9 | 5 | 10 |
| 2L + 4L | 3 |  | 17 | 3 | 0 | 0 | 0 | 4 | 0 | 2 | 4 | 2 | 14 |

Cells are color-coded according to the proportion (in percentages) of patients with metastases in each lymph node station, ranging from green (low prevalence) to red (high prevalence). Grey-shaded cells indicate that fewer than 10 patients were included in the respective subgroup. Aggregated regional categories (e.g., all abdominal, upper mediastinal) represent summary groupings and are consequently left uncolored in the table. * Percentages may appear high in small subgroups.

**Table S3.** Prevalence of lymph node metastases (LNM) per station and clinical subgroup in patients with esophageal squamous cell carcinoma.

|  | **Histology** | **Location** | | | | **Cliniacl T-stage** | | | | **Clincial N-stage** | | **Neoadjuvant** | |
| --- | --- | --- | --- | --- | --- | --- | --- | --- | --- | --- | --- | --- | --- |
|  | SCC | Prox | Mid | Dist | GEJ | cT1 | cT2 | cT3 | cT4 | cN0 | cN+ | Yes | No |
| No. Of patients | n=55 | n=5 | n=24 | n=24 | n=2 | n=2 | n=9 | n=24 | n=20 | n=24 | n=31 | n=49 | n=6 |
| Total | 35 | 20 | 42 | 33 | 0 | 0 | 22 | 46 | 30 | 25 | 42 | 39 | 0 |
| **Intra-thoracic (AJCC)** |  |  |  |  |  |  |  |  |  |  |  |  |  |
| Thoracic duct | 4 | 0 | 7 | 0 | 0 | 0 | 0 | 9 | 0 | 11 | 0 | 4 | 0 |
| *Upper Mediastinal* | 4 | 0 | 5 | 5 | 0 | 0 | 0 | 5 | 5 | 0 | 7 | 4 | 0 |
| 2R | 4 | 0 | 14 | 0 | 0 | 0 | 0 | 0 | 11 | 0 | 6 | 4 | 0 |
| 2L | 0 | 0 | 0 | 0 | 0 | 0 | 0 | 0 | 0 | 0 | 0 | 0 | 0 |
| 4R | 0 | 0 | 0 | 0 | 0 | 0 | 0 | 0 | 0 | 0 | 0 | 0 | 0 |
| 4L | 4 | 0 | 0 | 9 | 0 | 0 | 0 | 13 | 0 | 0 | 8 | 5 | 0 |
| 5 | 0 | 0 | 0 | 0 | 0 | 0 | 0 | 0 | 0 | 0 | 0 | 0 | 0 |
| *Middle mediastinal* | 13 | 0 | 14 | 14 | 0 | 0 | 13 | 14 | 12 | 9 | 15 | 14 | 0 |
| 7 | 10 | 0 | 6 | 15 | 0 | 0 | 0 | 11 | 15 | 0 | 19 | 11 | 0 |
| 8M | 13 | 0 | 15 | 13 | 0 | 0 | 20 | 29 | 0 | 18 | 8 | 16 | 0 |
| *Lower mediastinal* | 7 | 50 | 6 | 0 | 0 | 0 | 0 | 0 | 15 | 8 | 6 | 7 | 0 |
| 8L | 7 | 50 | 6 | 0 | 0 | 0 | 0 | 0 | 15 | 8 | 7 | 8 | 0 |
| 9 | 0 | 0 | 0 | 0 | 0 | 0 | 0 | 0 | 0 | 0 | 0 | 0 | 0 |
| **Abdominal (JGCA)** | 24 | 25 | 27 | 23 | 0 | 0 | 13 | 40 | 15 | 14 | 32 | 27 | 0 |
| 1 | 3 | 0 | 7 | 0 | 0 | 0 | 0 | 9 | 0 | 0 | 6 | 4 | 0 |
| 2 | 9 | 0 | 11 | 10 | 0 | 0 | 0 | 27 | 0 | 6 | 13 | 11 | 0 |
| 3 | 5 | 0 | 0 | 12 | 0 | 0 | 0 | 13 | 0 | 0 | 9 | 6 | 0 |
| 7 | 12 | 33 | 10 | 11 | 0 | 0 | 14 | 13 | 11 | 5 | 17 | 13 | 0 |
| 8 | 0 | 0 | 0 | 0 | 0 | 0 | 0 | 0 | 0 | 0 | 0 | 0 | 0 |
| 9 | 6 | 0 | 14 | 0 | 0 | 0 | 0 | 7 | 11 | 7 | 5 | 7 | 0 |
| 11p | 3 | 0 | 7 | 0 | 0 | 0 | 0 | 10 | 0 | 0 | 7 | 4 | 0 |
| **Paratracheal** |  |  |  |  |  |  |  |  |  |  |  |  |  |
| 2R + 4R | 2 | 0 | 5 | 0 | 0 | 0 | 0 | 0 | 5 | 0 | 3 | 2 | 0 |
| 2L + 4L | 3 | 0 | 0 | 6 | 0 | 0 | 0 | 7 | 0 | 0 | 5 | 3 | 0 |

Cells are color-coded according to the proportion (in percentages) of patients with metastases in each lymph node station, ranging from green (low prevalence) to red (high prevalence). Grey-shaded cells indicate that fewer than 9 patients were included in the respective subgroup. Aggregated regional categories (e.g., all abdominal, upper mediastinal) represent summary groupings and are consequently left uncolored in the table. * Percentages may appear high in small subgroups.

**Table S4.** Prevalence of lymph node metastases (LNM) per pathological staging.

|  | **Pathological T stage** | | | | |
| --- | --- | --- | --- | --- | --- |
| Total | (y)pT0 | (y)pT1 | (y)pT2 | (y)pT3 | (y)pT4 |
| *No. of Patients* | n=64 | n=40 | n=26 | n=61 | n=19 |
| *Patients with LNM* | 0% | 0% | 0% | 0% | 0% |
| **Intra-thoracic (AJCC)** |  |  |  |  |  |
| Thoracic duct | 6% | 4% | 9% | 16% | 9% |
| *Upper Mediastinal* | 4% | 0% | 10% | 9% | 17% |
| 2R | 4% | 0% | 0% | 12% | 11% |
| 2L | 0% | 0% | 0% | 8% | 0% |
| 4R | 4% | 0% | 5% | 2% | 6% |
| 4L | 0% | 0% | 10% | 6% | 0% |
| 5 | 0% | 0% | 0% | 6% | 0% |
| *Middle mediastinal* | 8% | 6% | 5% | 22% | 31% |
| 7 | 4% | 6% | 6% | 14% | 27% |
| 8M | 7% | 0% | 0% | 26% | 20% |
| *Lower mediastinal* | 3% | 0% | 9% | 21% | 43% |
| 8L | 4% | 0% | 10% | 16% | 40% |
| 9 | 0% | 0% | 0% | 15% | 33% |
| **Abdominal (JGCA)** | 18% | 31% | 30% | 52% | 28% |
| 1 | 0% | 15% | 13% | 28% | 40% |
| 2 | 3% | 10% | 0% | 21% | 33% |
| 3 | 0% | 13% | 0% | 16% | 8% |
| 7 | 10% | 22% | 25% | 33% | 21% |
| 8 | 0% | 3% | 0% | 2% | 6% |
| 9 | 10% | 3% | 17% | 16% | 10% |
| 11p | 6% | 0% | 0% | 21% | 0% |
| **Paratracheal** | 0% | 0% | 0% | 0% | 0% |
| 2R + 4R | 4% | 0% | 5% | 7% | 11% |
| 2L + 4L | 0% | 0% | 8% | 3% | 0% |

Cells are color-coded according to the proportion (in percentages) of patients with metastases in each lymph node station, ranging from green (low prevalence) to red (high prevalence). Aggregated regional categories (e.g., all abdominal, upper mediastinal) represent summary groupings and are consequently left uncolored in the table. * Percentages may appear high in small subgroups.
